# Unusual Closed Traumatic Avulsion of Both Flexor Tendons in Zones 1 and 3 of the Little Finger

**DOI:** 10.1155/2016/6837298

**Published:** 2016-08-30

**Authors:** Marie-Aimée Päivi Soro, Thierry Christen, Sébastien Durand

**Affiliations:** Department of Plastic and Hand Surgery, Lausanne University Hospital, rue du Bugnon 46, 1011 Lausanne, Switzerland

## Abstract

Closed tendon avulsion of both flexor tendons in the same finger is an extremely rare condition. We encountered the case of a patient who presented a rupture of the flexor digitorum profundus in zone 1 and flexor digitorum superficialis in zone 3 in the little finger. This occurrence has not been reported previously. We hereby present our case, make a review of the literature of avulsion of both flexor tendons of the same finger, and propose a treatment according to the site of the ruptures.

## 1. Introduction

Closed tendon avulsion is a well recognized injury in hand surgery. Also called* Jersey finger*, it usually involves the flexor digitorum profundus (FDP) tendon. The typical mechanism is a forced hyperextension on a fully flexed finger. It is often encountered in contact sports like rugby, American football, or judo when a player grabs his opponent's shirt with the tip of his finger while the opponent is running away.

Closed avulsion of the flexor digitorum superficialis (FDS) associated with an avulsion of the FDP is a rare occurrence. Only 8 cases have been reported since 1984 [1–9].

We encountered an unusual case of closed avulsion of both flexor tendons of the little finger with a rupture of the FDP in zone 1 and FDS in zone 3. Combination of simultaneous avulsion in zones 1 and 3 has not been reported previously. We will present our case, review the literature, and propose a treatment according to the site of the ruptures.

## 2. Case Report

**Figure 1:**
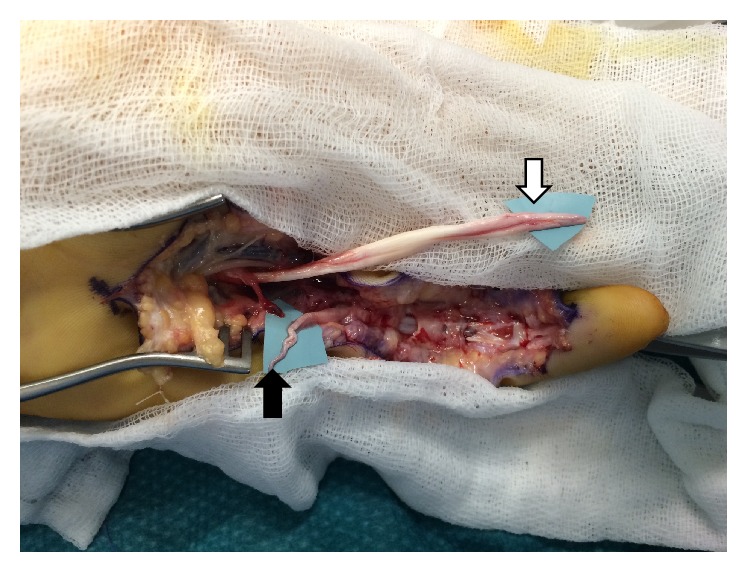
The white arrow indicates the proximal stump of the FDP, and the black arrow the distal dilacerated stump of the FDS.

A 30-year-old patient presented to the emergency unit after a rugby game with the impossibility to flex his fifth finger on the left hand. Surgical exploration was performed the same day; it demonstrated a rupture of the FDP a few millimetres from its insertion and a laceration of the FDS in the mid-palm (Figure 1). No previous trauma or other pathology could explain this double rupture.

The FDP was reinserted using a pull-out technique. In order to avoid flexion deformity of the distal interphalangeal (DIP) joint related to excessive tension of the FDP, the procedure was completed with a lengthening Z-plasty of the FDP in the forearm proximal to the carpal tunnel.

As the rupture of the FDS occurred in the mid-palm, a reconstruction would not interfere with the course of the FDP in the digital canal. Therefore the suture of the FDS was reinforced with a palmaris longus tendon graft.

The patient was immobilized in a Duran splint and underwent the usual rehabilitation protocol for flexor tendon lesions with physical therapists.

**Figure 2:**
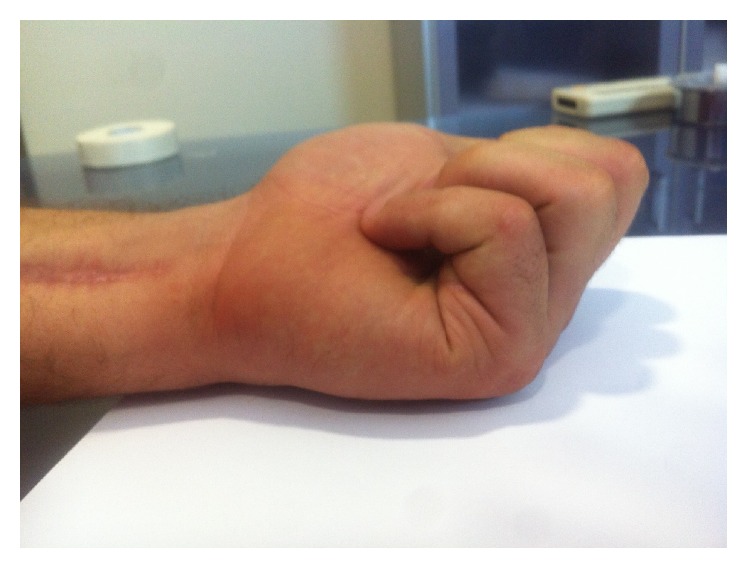
6 months after surgery.

**Figure 3:**
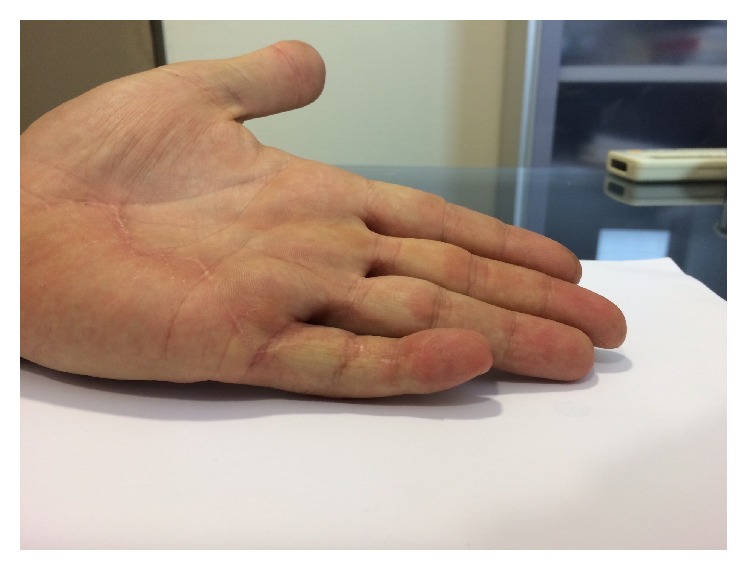
6 months after surgery.

Follow-up at 6 months showed the patient was able to touch his palm with his little finger (Figures 2 and 3) and return to work. The mobility of the finger in extension/flexion was as follows: metacarpophalangeal (MCP) joint: 0/0/95°, proximal interphalangeal (PIP) joint: 0/10/85°, and distal interphalangeal (DIP) joint: 0/10/25°. Active flexion of the PIP joint was observed when action of the FDP was prevented.

## 3. Discussion-Review of the Literature

We found 8 case reports (11 fingers) of closed avulsion of both flexor tendons in a single finger [1–9]. The characteristics of the patients, injury type, surgical repair technique, and results are summarized in Table 1.

The mean age of the patients was 26.9 years. They were all men. The dominant hand was affected in 42% of patients (three authors did not mention the hand dominance). The ring finger (5/11 fingers) was the most commonly affected. Six different mechanisms of injury were encountered. Two patients presented with a typical Jersey finger [3, 8]. Two others suffered a blast injury [2, 9] and had several fingers affected. Two patients had a mechanism of traction-hyperextension [1, 4, 5]. The patient from Naohito et al. [7] hurt his fifth finger in a fall without further details. Repeated microtraumas were responsible for the rupture in one case [6]. The mean interval between traumatism and surgical exploration was 7.8 days (median 3 days). Five patients underwent surgery in the first week after trauma and 3 patients after 2 weeks.

One author used ultrasonography as a diagnostic help [9]. Their localisation of the rupture was accurate.

All FDS tendons were ruptured in zone 2 except in our case (zone 3).

All FDP including our case were ruptured in zone 1 except for two in zone 2 [6, 7].

Six authors chose to resect the FDS [1, 4–9]. In the other two cases [2, 3], the rupture was not intratendinous but at the insertion and associated with a bony avulsion. Both authors reinserted it with a transosseous suture.

Four authors reinserted the FDP through a pull-out suture [2, 3, 8, 9]. Naohito et al. [7] performed an end-to-end suture for a zone 2 rupture. Three authors [1, 4–6] performed an FDP resection followed by a tendon graft in a one- or two-stage procedure when rupture was in zone 2 or in cases of delayed presentation.

The outcome was reported as good to excellent in 4 cases out of 8. In the other four cases, the results were poorer due to an FDP lesion in zone 2 or dilacerated in zone 1. Seven out of nine patients went back to work with no or little disability [1, 3–7, 9]. Two authors did not mention whether their patients were able to return to normal activity [2, 8].

Our case is unusual as the FDS was dilacerated in zone 3. The literature shows that few patients (2 out of 8) benefited from an FDS repair [2, 3]. This is probably due to the fact that both flexor tendon sutures in zone 2 are known to result in poorer outcome than more proximal lesions [10]. The rupture in zone 3 allowed for an FDS suture reinforced by a tendon graft. The other distinctive characteristic that our case presents is that the FDP pull-out suture was completed with a lengthening Z-plasty in the forearm which prevented tension deformity from the DIP and PIP joint. We were able to repair both tendons and avoid a suture in zone 2. The result was good at the 6-month follow-up visit and the patient was able to return to work.

Only one author used ultrasonography as a diagnostic tool before surgery [9]. Its utility has been shown as a diagnostic help for flexor tendon injuries [11, 12]. Double flexor tendon injury in the same finger is a rare occurrence and the usefulness of ultrasonography still needs to be explored in this indication. However, it might be an interesting tool to assess the zones in which the tendons have ruptured in order to define preoperatively surgical strategy.

In conclusion:

1. When FDS is ruptured in zone 3 and FDP in zone 1, an FDS repair is recommended as it will not impair the FDP function in the digital canal and reinforces the action of the FDP. The FDP can benefit from a pull-out suture. If the FDP is dilacerated and the pull-out suture prevents full extension of the DIP or PIP joint we recommend a lengthening Z-plasty in the forearm so as to avoid a resection-graft which has worse outcomes.
2. When the FDS is ruptured in zone 2 and the FDP in zone 1, we recommend to reinsert the FDP with a pull-out suture. An FDS reinsertion could be considered in particular conditions [2, 3].
3. If both tendons are ruptured in zone 2, whenever possible, both tendons should be repaired, as long as the FDP is gliding freely. If the tendons are severely dilacerated or oedematous and the FDS suture impairs the FDP course in the digital canal, the FDS should be resected [10]. The FDP, depending on the dilacerations and extension of the lesions, could be either sutured or grafted [6, 7]. Another surgical option is the transfer of a hemi-FDP-tendon from an adjacent finger [13].

## Figures and Tables

**Table 1:** Closed avulsion of both flexors in the same finger.

Author	Patient age, sex, HD	Mechanism	Finger(s)	Site of rupture	Days before surgery	Technique	Months of postsurgery follow-up	Success
Our case	30, ♂, R	Jersey finger	D5 L	FDS: zone 3 FDP: zone 1	0	FDP: pull-out, FDS: suture reinforced by a tendon graft	6	E/F MCP: 0/0/95°, PIP: 0/10/85° DIP: 0/10/22°. Back to work.

Cheung and Chow [3]	24, ♂, R	Jersey finger	D4 R	FDS: zone 2 FDP: zone 1	4	Both tendons sutured to a periosteal flap and FDP reinforced by pull-out	3.5	Full range of motion MP + PIP, flexum 4° DIP. Back to work.

Oğün et al. [8]	21, ♂, NM	Jersey finger	D4 R	FDS: zone 2 FDP: zone 1	0	FDP: pull-out, FDS: resected	19	Total active range of motion = 230°, flexum PIP, DIP stiffness.

Naohito et al. [7]	49, ♂, L	Direct shock	D5 R	FDS: zone 2 FDP: zone 2	20	FDP: end-to-end suture, FDS resected	4	E/F: MCP: 30/0/80; PIP: 0/40/85, DIP: 0/5/60. Back to work.

Matthews and Walton [6]	28, ♂, R	Repeated microtrauma	D3 R	FDS: zone 2 mi-P1 FDP: zone 2 mi-P1	14	Two-stage repair: resection, silicone rod, reoperation at 10 weeks, palmaris longus graft.	3.5	Good result: normal flexion PIP, DIP stiffness. Back to work.

Cañadas Moreno et al. [2]	16, ♂, NM	Blast	D2 L: FDP + FDS D3 L: FDP + FDS D4 + D5: FDP	FDS: zone 2 FDP: zone 1	0	4 FDP: pull-out, 2 FDS: anchor suture technique	4 months	IPD: flexum 30° D2, D3, D5
							3 years	Completely recovered.

Toussaint et al. [9]	23, ♂, R	Blast	D4 L D5 L	FDS: zone 2 FDP dilacerated in zone 1 + volar plate pull-out	D1	FDP: pull-out, FDS: resected	7	D4: PID: flexum 15°, PIP: flexum 10°. D5: PID flexum 40°, PIP flexum 10°. Back to work.

Backe and Posner [1]	23, ♂, NM	Traction-hyperextension	D4 R	FDS: zone 2 FDP: zone 1	4 weeks	Palmaris longus tendon graft	NM	Complete extension and active flexion to within 1.5 cm of the midpalmar crease. DIP stiffness. Back to work as truck driver.

Lanzetta and Conolly [4, 5]	28, ♂, R	Traction-hyperextension	D4 R	FDS: zone 2 FDP: zone 1	3	Two-stage repair: excision of both tendons, left plantaris tendon graft 9 weeks after the 1st surgery	4	Recovery of full extension and flexion. Back to work as a mechanic 4 months after 2nd surgery.

HD = hand dominance, R = right, L = left, NM = not mentioned, E/F = extension/flexion, MCP = metacarpophalangeal joint, PIP = proximal interphalangeal joint, and DIP = distal interphalangeal joint.
